# Acute gastrointestinal injury severity assessment informs nutritional support and prognostic evaluation in critically ill children: a narrative review

**DOI:** 10.3389/fnut.2026.1911973

**Published:** 2026-08-14

**Authors:** Cizheng Zeng, Tufeng Li, Jiayuan Wu, Shiwen Rong, Xiaohua Liu, Yinhui Chen, Nali Cai, Limei Zhang, Wen Li, Ling Liu

**Affiliations:** 1 Pediatric Intensive Care Unit, Affiliated Hospital of Guangdong Medical University, Zhanjiang, China; 2 Department of Scientific Research, Affiliated Hospital of Guangdong Medical University, Zhanjiang, China

**Keywords:** critical illness, enteral feeding, feeding intolerance, gastric residual volume, intra-abdominal hypertension, multiple organ dysfunction, nutritional support, pediatric intensive care

## Abstract

Acute gastrointestinal injury (AGI) affects 41–65% of children admitted to pediatric intensive care units (PICUs) and is associated with prolonged mechanical ventilation, extended hospital stays, multiple organ dysfunction, and increased mortality. Although the 2012 European Society of Intensive Care Medicine (ESICM) AGI grading system provides standardized gastrointestinal assessment for adults, no validated pediatric-specific tool currently exists. This narrative review critically synthesizes the prognostic and nutritional relevance of AGI severity assessment in critically ill children. A structured literature search of PubMed, Embase, Web of Science, Google Scholar, Cochrane Library, CINAHL, CNKI, and Wanfang Data through December 2025 yielded 78 peer-reviewed articles, guidelines, and consensus statements. Two reviewers independently screened records; evidence was appraised narratively with explicit attention to study design and methodological quality, assigning greater weight to multicenter prospective studies, pediatric guidelines, and meta-analyses (level I–II evidence). No formal quality assessment, consensus methodology, or systematic evidence synthesis was performed, consistent with narrative review methodology. Existing pediatric adaptations of adult AGI criteria have been reported in single-center and observational studies and include reported thresholds for gastric residual volume (>5 mL/kg), intra-abdominal pressure gradations, and feeding intolerance definitions. Higher AGI severity is consistently associated with increased mortality, prolonged mechanical ventilation, and greater multiple organ dysfunction syndrome. Synthesis of AGI severity concepts with pediatric nutrition guidelines suggests that early enteral nutrition appears feasible in mild dysfunction, whereas severe grades may warrant cautious parenteral supplementation with dynamic reassessment. This review identifies critical gaps: the absence of multicenter validation, the subjective nature of grading variables, and the lack of age-stratified nutritional trials. In conclusion, AGI severity assessment holds conceptual promise for risk stratification and nutritional guidance in pediatric intensive care, but requires formal consensus methodology, systematic evidence synthesis, and prospective validation before clinical adoption.

## Graphical abstract

**Figure:**
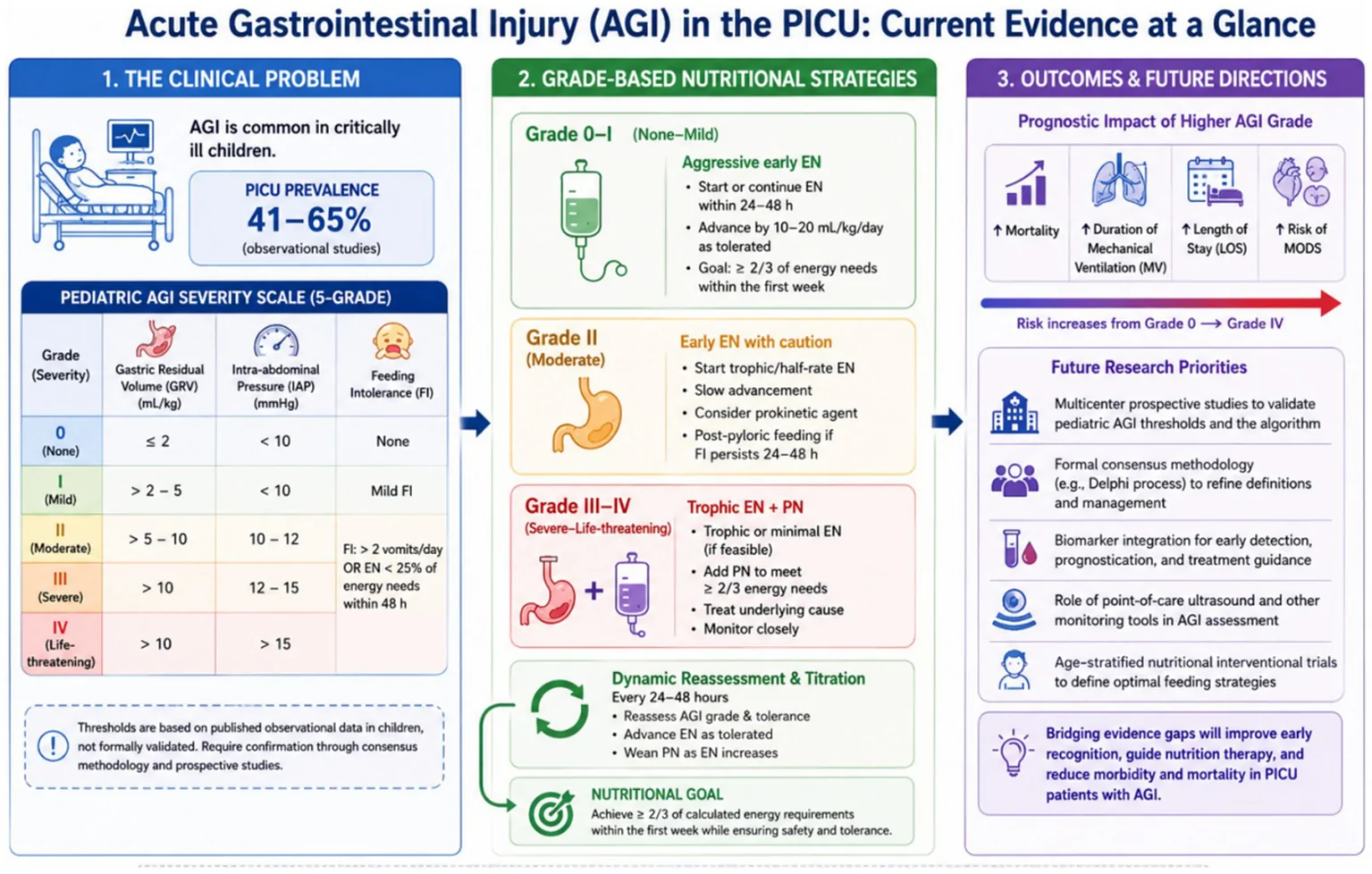
Simplified visual summary of acute gastrointestinal injury (AGI) severity assessment concepts for the pediatric intensive care unit (PICU). The left panel depicts the clinical problem (41–65% PICU prevalence) and the spectrum of pediatric AGI severity with age-specific thresholds for gastric residual volume (GRV), intra-abdominal pressure (IAP), and feeding intolerance (FI) derived from existing literature. The central panel illustrates severity-based nutritional strategies synthesized from current guidelines: aggressive early enteral nutrition (EN) for mild dysfunction to preserve gut integrity, and trophic EN combined with parenteral nutrition (PN) for severe grades, with dynamic reassessment and titration every 24–48 h. The right panel summarizes prognostic outcomes [mortality, mechanical ventilation (MV), length of stay (LOS), multiple organ dysfunction syndrome (MODS)] and future research priorities, including multicenter randomized controlled trials (RCTs), biomarker integration, and age-stratified nutritional validation.

## 1 Introduction

The acute gastrointestinal injury (AGI) grading system, introduced by the European Society of Intensive Care Medicine (ESICM) in 2012, offers a standardized framework for assessing gastrointestinal dysfunction in critically ill adults (1). It categorizes AGI severity from grade 0 (no dysfunction) to grade IV (life-threatening failure with remote organ dysfunction), using bedside markers such as feeding intolerance and elevated gastric residuals. In children, gastrointestinal dysfunction has more recently been defined by the Pediatric Organ Dysfunction Information Update Mandate (PODIUM) consensus conference, which proposed pediatric-specific gastrointestinal dysfunction criteria within a multi-organ dysfunction framework (2, 3). The PODIUM criteria and the AGI grading concept are complementary rather than interchangeable: the former situates gastrointestinal dysfunction within organ-failure scoring, whereas the latter grades gastrointestinal severity itself.

In the pediatric intensive care unit (PICU), AGI affects 41–65% of critically ill children (4–6), with escalating severity linked to worse outcomes, including longer intensive care unit and hospital lengths of stay, prolonged mechanical ventilation, heightened multiorgan failure risk, and greater mortality (5, 7, 8). The grading concept also informs nutritional management; early enteral nutrition is recommended for children with mild AGI to maintain gut integrity, while those with severe dysfunction often require cautious parenteral nutrition to optimize caloric intake and minimize complications (9–11). Despite its established utility in adults, the lack of a validated pediatric-specific AGI severity tool remains a critical gap.

This review differs from previous syntheses in three respects. First, rather than examining feeding intolerance definitions (12, 13), feeding intolerance measurement (14), gastrointestinal dysfunction criteria (2), or nutrition guidelines (15, 16) in isolation, it integrates all three around the AGI severity construct, linking grading parameters to both prognostic outcomes and grade-specific nutritional strategy. Second, it incorporates emerging evidence unavailable to earlier reviews, including dynamic AGI trajectory phenotyping (6) and the GASTRIC-PICU randomized trial (17), which directly challenges the gastric residual volume thresholds on which earlier grading proposals relied. Third, it applies an explicit evidence hierarchy and certainty appraisal, distinguishing validated knowledge from observationally derived proposals—an appraisal we consider essential before pediatric AGI grading proceeds toward consensus standardization.

This narrative review critically synthesizes evidence on AGI severity assessment in PICU patients, examines existing pediatric adaptations of adult criteria reported in the literature, discusses their prognostic and nutritional implications, and identifies key gaps to guide prospective investigations. Terminology: throughout this review, “feeding intolerance” denotes the clinical syndrome of failure to achieve enteral nutrition targets accompanied by gastrointestinal symptoms; “gastrointestinal dysfunction” denotes the broader physiological construct, consistent with PODIUM usage; and “AGI severity” or “AGI grade” refers to the ESICM-derived grading concept. This integration—linking severity grading, prognostic evaluation, and nutritional management—has not been comprehensively synthesized previously and addresses an important gap in pediatric critical care nutrition.

## 2 Methods

This narrative review employed a systematic literature search strategy to synthesize evidence on AGI severity assessment in pediatric critical care. A narrative review methodology was selected because the available evidence comprises heterogeneous study designs (observational cohorts, consensus statements, and guideline recommendations) without sufficient homogeneity for formal meta-analysis or systematic evidence synthesis. Databases searched included PubMed, Embase, Web of Science, Google Scholar, Cochrane Library, CINAHL, CNKI, and Wanfang Data, covering publications from inception to December 2025. The addition of Cochrane Library and CINAHL enhances coverage of systematic reviews and nursing-focused literature relevant to pediatric critical care nutrition and gastrointestinal assessment. Search terms encompassed “acute gastrointestinal injury,” “AGI grading,” “pediatric intensive care,” “prognosis,” “nutritional guidance,” and equivalents in English and Chinese. No language restrictions were applied; non-English articles with relevant pediatric data were included and translated by bilingual reviewers. Inclusion criteria prioritized peer-reviewed articles, guidelines, and consensus statements on AGI pathophysiology, pediatric adaptations, prognostic outcomes, nutritional strategies, and implementation challenges in children aged 0–18 years. Exclusion encompassed nonhuman studies, adult-only data without pediatric relevance, conference abstracts, and low-quality reports. Two reviewers independently screened titles and abstracts, then full texts, resolving discrepancies via consensus with a third reviewer. The literature selection process is summarized in a PRISMA-style flow diagram. No formal quality assessment, systematic evidence synthesis, meta-analysis, or consensus methodology was performed, consistent with narrative review methodology; however, evidence was appraised narratively with explicit attention to study design and methodological quality. Greater weight was assigned to multicenter prospective studies (level II evidence according to the Oxford Center for Evidence-Based Medicine hierarchy), pediatric-specific guidelines (level IV), and meta-analyses (level I) when formulating considerations. Single-center retrospective studies (level III) and expert opinion (level V) were interpreted with appropriate caution (Table 1). This approach aligns with narrative review methodology while providing readers with transparent guidance regarding the certainty underlying individual recommendations. This review does not propose a new clinical framework; rather, it critically summarizes existing pediatric adaptations and identifies prerequisites for formal validation.

**Table 1:** Nutritional support strategies for critically ill children with acute gastrointestinal injury: a synthesis of existing guideline recommendations.

Grade	AGI severity	Nutritional strategy	Enteral nutrition approach	Parenteral nutrition approach	Energy and protein targets	Monitoring parameters	Complications to screen	Guideline alignment
0	Normal function	Aggressive early EN to preserve gut integrity and prevent dysfunction	Initiate within 24–48 h of admission if hemodynamically stable; advance to full caloric target rapidly; standard polymeric formula; gastric feeding preferred	Not required unless contraindicated to EN	100% of calculated requirements by day 3–5; protein 1.5–2.5 g/kg/day depending on age	GRV every 4–6 h initially; tolerance assessment; stool pattern; abdominal examination	Refeeding syndrome; hyperglycemia; aspiration risk during initiation	ASPEN/SCCM: early EN initiation; ESPNIC: advance to target within 3–5 days
I–II	Risk/Mild dysfunction	Aggressive EN with careful monitoring; maintain gut integrity while addressing early FI	Continue or initiate EN within 24 h; advance to caloric target over 3–5 days; consider semi-elemental formula if intolerance; prokinetics if GRV > 5 mL/kg; continuous infusion may improve tolerance	Minimal or supplemental PN only if EN < 50% target after 3–5 days; avoid overfeeding	100% calculated requirements by day 5–7; protein 1.5–2.5 g/kg/day; adjust for stress/metabolic demand	GRV every 4–6 h (ultrasound preferred over aspiration); IAP if distension present; daily weight; nitrogen balance; serum prealbumin	Aspiration; necrotizing enterocolitis (especially neonates/infants); feed intolerance progression; hyperglycemia	ASPEN/SCCM: monitor FI, optimize protein; ESPNIC: tailored nutrition based on GI symptom severity; SRLF/GFRUP: individualized approaches
III	GI failure	Trophic EN combined with PN to meet nutritional needs while allowing GI rest	Trophic feeds at 10–20% of target or 0.5–1 mL/kg/h continuous; minimal enteral nutrition to maintain mucosal integrity; post-pyloric feeding if gastric intolerance; hold EN if hemodynamic instability or ACS	Initiate PN within 24–48 h if EN < 25% target; provide 80–100% calculated requirements via PN; include glutamine where available; monitor for PN-associated liver disease	Total 100% requirements via combined EN + PN; protein 2.0–3.0 g/kg/day; adjust based on catabolic state	GRV every 6 h or post-pyloric assessment; daily IAP; abdominal girth (despite poor reliability); GI symptoms; liver function; triglycerides; glucose	PN-related cholestasis; hyperglycemia; catheter-related infection; intestinal atrophy from lack of EN; bacterial translocation; ACS progression	ESPNIC: PN when EN insufficient; SRLF/GFRUP: cautious progression; ASPEN/SCCM: avoid underfeeding
IV	Life-threatening failure	Minimal trophic EN with full PN support; prioritize hemodynamic stability and ACS management	Minimal trophic EN (5–10% target) only if hemodynamically stable and no ACS; otherwise nil per os; consider mucosal protectants; no advancement until grade improves	Full PN providing 100% calculated requirements; aggressive protein delivery 2.5–3.5 g/kg/day; monitor closely for metabolic complications; cyclical PN if hepatic dysfunction	100% requirements via PN; protein 2.5–3.5 g/kg/day; micronutrient supplementation; consider immunonutrition formulas once grade improves	Hourly hemodynamic monitoring; IAP every 4–6 h; ACS surveillance; lactate; organ perfusion markers; strict glucose control; liver function	Severe ACS requiring decompression; bowel ischemia/necrosis; gut-origin sepsis; PN-associated liver disease; immune dysfunction from lack of EN; multiple organ failure	ASPEN/SCCM: PN when EN contraindicated; ESPNIC: GI rest in severe failure; SRLF/GFRUP: dynamic reassessment; WSACS: ACS management protocols

This table synthesizes grade-specific nutritional interventions derived from ASPEN/SCCM, ESPNIC, and SRLF/GFRUP guideline recommendations, contextualized within the spectrum of GI dysfunction severity observed in pediatric critical care. Operational definitions are based on existing evidence and require validation.

AGI, acute gastrointestinal injury; EN, enteral nutrition; PN, parenteral nutrition; FI, feeding intolerance; IAH, intra-abdominal hypertension; GI, gastrointestinal; GRV, gastric residual volume.

## 3 Results

### 3.1 Pathophysiological mechanisms of AGI in critically ill children

In critically ill children, AGI emerges from multifactorial insults impairing intestinal motility, nutrient uptake, and mucosal barrier integrity, further intensified by age-related factors such as reduced epithelial thickness and immature immune maturation (2, 18). Core drivers—ischemia–reperfusion injury, cytokine-driven inflammatory cascades, microbial dysbiosis, and perturbed neuro-endocrine-immune signaling—form self-reinforcing loops that propagate distant organ dysfunction (19).

Intestinal hypoperfusion, especially during shock or sepsis, is a critical trigger for AGI. Sympathetic vasoconstriction diverts blood from the gut, causing mucosal ischemia; reperfusion then generates reactive oxygen species, intensifying lipid peroxidation and disrupting tight junctions (20, 21). Limited physiological reserves and elevated metabolic demands amplify villous injury in children, increasing mucosal permeability and driving systemic inflammation (22). The adult grading system delineates reversible phases, guiding timely resuscitation to prevent progression to severe dysfunction.

The pediatric gut barrier consists of a physical layer (comprising mucus and epithelial cells) and an immunological layer (including gut-associated lymphoid tissue and secretory immunoglobulin A). Critical illness activates pro-inflammatory pathways: tumor necrosis factor-alpha and interleukin-6 promote endothelial activation, leukocyte infiltration, and enterocyte apoptosis, disrupting tight junction proteins (zonula occludens-1, occludin). The resulting “leaky gut” permits translocation of bacteria and pathogen-associated molecular patterns into the portal circulation and mesenteric lymphatics, perpetuating a self-sustaining cycle of systemic inflammation and multiple organ dysfunction syndrome (MODS) (23). Accordingly, severe grades conceptually support anti-inflammatory nutritional strategies—early enteral nutrition with immune-modulating formulas, or cautious parenteral nutrition—to mitigate inflammation and preserve barrier integrity (9, 19).

Stressors like antibiotics, ischemia, or hypoperfusion disrupt the microbiota, depleting commensals such as Bifidobacterium and Lactobacillus, while enabling proliferation of pathogens such as *Klebsiella pneumoniae*, *Escherichia coli*, and Clostridioides difficile (24, 25). These organisms shed lipopolysaccharides that traverse the compromised barrier, contributing to sepsis and MODS (23). Metagenomic studies link such dysbiotic shifts—reduced microbial diversity and pathobiont overgrowth—to amplified barrier dysfunction and systemic inflammation, offering candidate biomarkers for early AGI detection and progression monitoring (26).

**Figure 1:**
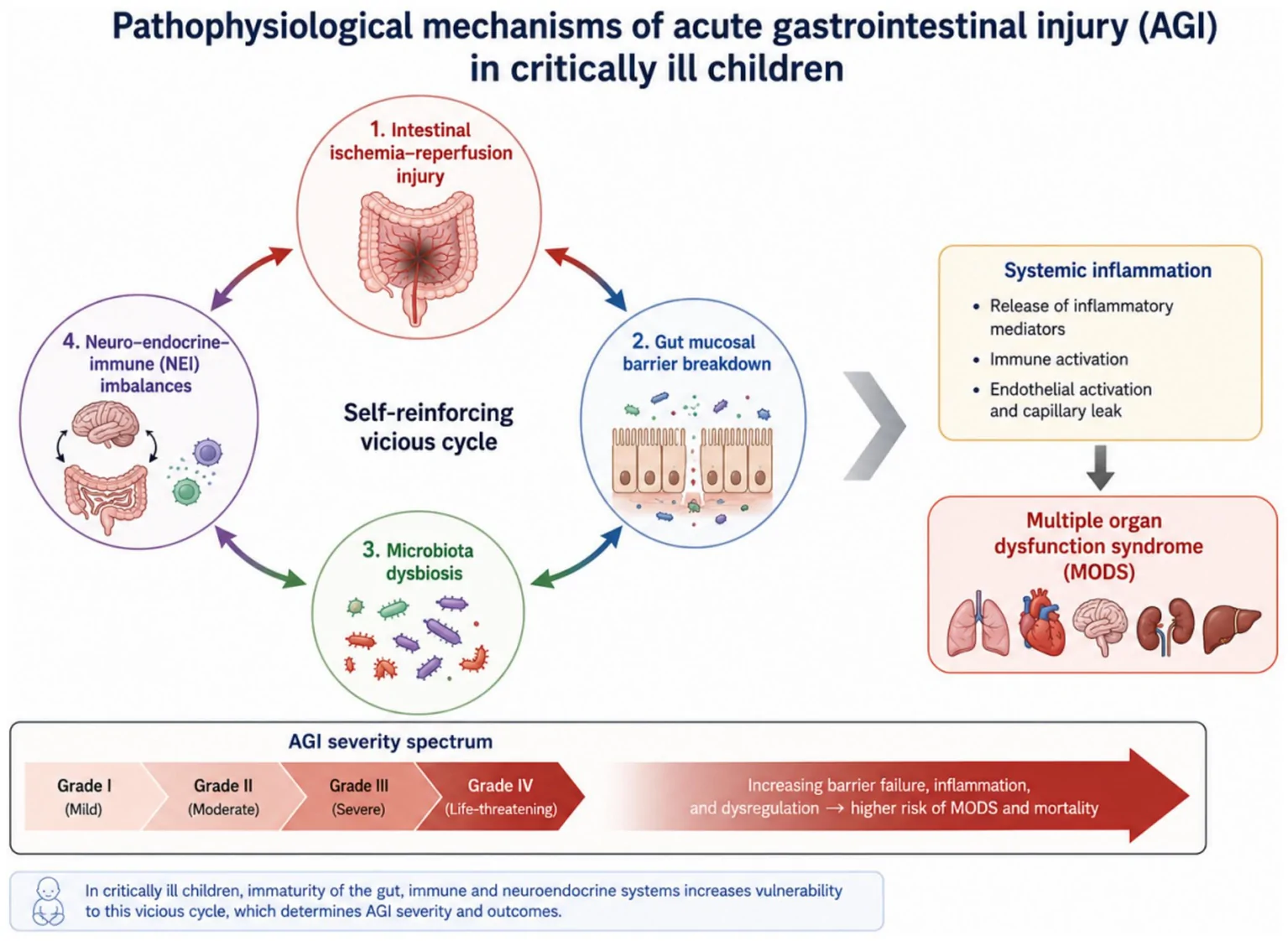
Pathophysiological mechanisms of acute gastrointestinal injury (AGI) in critically ill children. The schematic depicts four interconnected processes, including intestinal ischemia–reperfusion injury, gut mucosal barrier breakdown, microbiota dysbiosis, and Neuro-endocrine-immune (NEI) imbalances, and their bidirectional, self-reinforcing relationships that propagate systemic inflammation and Multiple Organ Dysfunction Syndrome (MODS).

Neuro-endocrine-immune imbalances further shape AGI severity. Stress-induced sympathetic hyperactivity elevates norepinephrine, inhibiting peristalsis and increasing intestinal permeability (Figure 1), while hypothalamic–pituitary–adrenal overactivation raises cortisol, hindering epithelial regeneration (27). Endocrine perturbations—elevated cholecystokinin, peptide YY, and glucagon-like peptide-1 with reduced ghrelin and motilin—are associated with delayed gastric emptying and enteral intolerance (28). Pediatric immaturity intensifies these effects, and the resulting dysregulation impedes nutritional tolerance, informing severity-based interventions (2, 27).

### 3.2 Adult AGI grading and emerging pediatric considerations

The ESICM AGI grading system standardizes gastrointestinal dysfunction assessment in critically ill adults, categorizing severity into five grades (0–IV) from normal function to life-threatening failure impacting distant organs. Key indicators include feeding intolerance, diarrhea, abdominal distension, gastric residual volume, and intra-abdominal pressure, enabling consistent monitoring and prognostic evaluation (1). Whereas the PODIUM criteria classify gastrointestinal dysfunction within a multi-organ scoring framework, the AGI concept grades gastrointestinal severity itself; the two systems are complementary rather than interchangeable (2). Application to critically ill children requires careful consideration of unique developmental physiology. Immature gastrointestinal motility impairs feeding tolerance; higher metabolic rates accelerate energy depletion; smaller gastric capacity increases residuals and vomiting; and immature immunity heightens infection susceptibility from barrier dysfunction (29). Consequently, pediatric AGI assessment necessitates age-specific considerations rather than direct adult extrapolation.

Feeding intolerance, defined as inability to achieve target enteral nutrition intakes combined with gastrointestinal symptoms including vomiting, diarrhea, or distention (12, 13), serves as a key marker in pediatric populations. Normal or low gastric residual volume indicates effective emptying, tolerance, reduced aspiration risk, and nutritional progress, enabling advancement (15, 30). Despite debates regarding its predictive value, most clinicians still use elevated gastric residual volume to diagnose feeding intolerance. However, the recently published GASTRIC-PICU Trial (17) directly tested whether routine GRV monitoring is necessary in mechanically ventilated children. This multicenter randomized trial found that not routinely measuring GRV was non-inferior to every-6-h monitoring for the primary outcome and significantly improved energy delivery. These findings fundamentally challenge the clinical utility of GRV-based grading thresholds (>5 mL/kg) proposed in this review and elsewhere. The trial suggests that routine GRV measurement may be unnecessary for safe enteral nutrition in mechanically ventilated children, challenging its role as a core component of pediatric AGI severity assessment. However, trial exclusions (preterm neonates, post-pyloric feeding, recent gut pathology or surgery, active gastrointestinal bleeding, necrotizing enterocolitis) nonetheless limit extrapolation to children with established high-grade AGI, in whom clinically indicated—rather than scheduled—gastric assessment may still inform care. Subsequently, pediatric thresholds differ from adult standards (200–500 mL) due to smaller capacity, requiring absolute (mL/kg) or percentage adjustments. Clinically, gastric residual volume exceeding 5 mL/kg has been reported to prompt feed reduction or prokinetics to prevent aspiration and necrotizing enterocolitis, though outcome correlations remain weak (14, 31). Ultrasound studies confirm that volumes greater than 5 mL/kg correspond to qualitative grade II dysfunction, predicting 95% limits (±1.65 mL/kg) for aspirated values (32). However, age stratification warrants explicit consideration. The same 5 mL/kg GRV cutoff has been applied from neonates to adolescents without validation in any age band. Neonates exhibit physiologically larger and more variable gastric residuals relative to feed volumes, horizontal gastric positioning with weaker lower esophageal sphincter tone, immature motility patterns, and heightened necrotizing enterocolitis susceptibility, all of which can mimic or amplify intolerance signs; infants show transitional physiology; and older children and adolescents increasingly approximate adult gastrointestinal function. Normal intra-abdominal pressure, feeding-tolerance patterns, and energy requirements likewise vary across developmental stages. Although GASTRIC-PICU stratified randomization by age group, it was not designed to validate age-specific thresholds (17). Age-stratified normative data are therefore a prerequisite for any future pediatric AGI tool.

Elevated intra-abdominal pressure critically determines AGI severity, directly impairing gastrointestinal perfusion and motility while escalating from risk to organ failure (33). The pediatric gold standard is transvesical measurement, instilling 1 mL/kg saline (maximum 25 mL) via Foley catheter at end-expiration in supine position. Normal intra-abdominal pressure in healthy children approximates 0 mmHg; in ventilated critically ill children it averages 7 ± 3 mmHg (range 4–10 mmHg), below adult baselines due to smaller abdominal volume and greater wall compliance (34, 35). Pediatric intra-abdominal hypertension has been described as sustained pressure exceeding 10 mmHg versus adult thresholds above 12 mmHg. Abdominal compartment syndrome is defined as sustained pressure exceeding 15 mmHg with organ dysfunction, aligning with evidence that persistent values between 10–15 mmHg correlate more strongly with adverse outcomes in children (34–36). Existing pediatric literature has suggested intra-abdominal pressure thresholds of 10–12 mmHg corresponding to grade II dysfunction, 12–15 mmHg to grade III, and sustained pressure exceeding 15 mmHg with organ failure to grade IV, consistent with abdominal compartment syndrome. These parameters require formal validation across age groups.

In critically ill children, vomiting, diarrhea, abdominal distention, bowel dilation, obstruction, and gastrointestinal bleeding are pivotal indicators that, accompanied by enteral nutrition failure, escalate AGI severity. Adult grade II defines feeding intolerance as failure to achieve less than 20 kcal/kg/day within 72 h (1). Pediatric modifications are warranted as age-specific metabolic variations and developmental energy requirements differ from adults. Integrating child-focused research, some investigators have utilized energy delivery thresholds, such as failure to achieve 25% of energy targets within 48 h, as markers of intolerance in pediatric populations (15, 37, 38). These thresholds require validation across age groups. In children, vomiting increases aspiration pneumonia risk, particularly when containing feed (13). In pediatric practice, vomiting thresholds lower than adult standards have been applied, with more than 2 episodes daily or regurgitation surpassing 50% of feed volume reported as markers of grade II dysfunction, reflecting smaller gastric capacity and aspiration vulnerability (12). Vomitus containing enteral feed indicates intolerance, demanding immediate nutrition interruption or prokinetic therapy, justifying gastrointestinal injury escalation if persistent. Diarrhea exceeding 4 loose stools daily or 20 mL/kg/day, excluding enteric infections, has been associated with grade II/III dysfunction in pediatric reports (12). Abdominal distention is noted without girth quantification; guidelines discourage serial circumference measurement due to poor reliability. Adult imaging criteria exist, while pediatric standards remain undefined. Table 2 summarizes the reported and proposed pediatric-specific parameters and thresholds identified in existing literature and current practice; as emphasized in the table footnote, none constitutes a validated pediatric standard.

**Table 2:** Pediatric considerations for acute gastrointestinal injury severity assessment: synthesis of existing evidence and current practice parameters.

Grade	Terminology	GI function	Gastric residual volume (GRV)	Intra-abdominal pressure (IAP)	Feeding intolerance (FI) criteria	Vomiting	Diarrhea	Abdominal distension	Other clinical indicators
0	Normal function	Normal GI function with no signs of dysfunction	Normal or low GRV; effective gastric emptying	Normal IAP in healthy children approximates 0 mmHg; in ventilated critically ill children averages 7 ± 3 mmHg (range 4–10 mmHg)	None; achieving enteral nutrition targets without GI symptoms	None or physiologic regurgitation	Normal stool pattern for age	None	Normal bowel sounds; no GI bleeding; no dilation on imaging
I	Risk of dysfunction	GI function partially impaired but no major impact on general condition; self-limiting condition	GRV may be mildly elevated but remains <5 mL/kg; trending upward from baseline	IAP may be intermittently elevated but remains <10 mmHg; transient increases without sustained hypertension	Failure to achieve enteral nutrition targets within expected time frame without overt GI symptoms; early warning signs	≤2 episodes per day; small volume regurgitation not containing feed	Mild increase in stool frequency but <4 loose stools per day and <20 mL/kg/day	Mild, transient distension without clinical significance; no serial girth measurement recommended	Absent or diminished bowel sounds; mild gastric paresis; no hemodynamic compromise
II	GI dysfunction	GI function clearly impaired and impacting general condition; requires specific intervention but not immediately life-threatening	GRV > 5 mL/kg prompting feed reduction or prokinetics; ultrasound confirms volumes >5 mL/kg correspond to qualitative grade II dysfunction with 95% limits (±1.65 mL/kg) for aspirated values	IAP 10–12 mmHg; sustained elevation above pediatric baseline (7 ± 3 mmHg) indicating intra-abdominal hypertension threshold lower than adult standard (>12 mmHg)	Failure to achieve ≥25% of calculated energy targets within 48 h; inability to advance EN despite prokinetics; clear GI symptoms present	>2 episodes per day or regurgitation surpassing 50% of feed volume; vomitus containing enteral feed indicates intolerance requiring immediate nutrition interruption or prokinetic therapy	≥4 loose stools per day or ≥20 mL/kg/day, excluding enteric infections; indicates grade II/III dysfunction in pediatric reports	Present but without girth quantification; guidelines discourage serial circumference measurement due to poor reliability; imaging may show mild bowel dilation	GI bleeding without hemodynamic compromise; absent bowel sounds; mild abdominal compartment symptoms; ileus on imaging
III	GI failure	GI function severely impaired and not responding to initial interventions; general condition significantly impacted; remote organ dysfunction may be present	Persistently elevated GRV > 5 mL/kg despite prokinetics and feed reduction; repeated aspiration required; high aspiration risk	IAP 12–15 mmHg; sustained intra-abdominal hypertension correlating more strongly with adverse outcomes in children than adult thresholds; persistent values in this range indicate significant perfusion impairment	Persistent enteral nutrition intolerance despite interventions; unable to achieve meaningful EN delivery; requirement for parenteral nutrition supplementation	Persistent vomiting despite nil per os status or minimal trophic feeds; vomiting with bile or blood	Severe diarrhea (>20 mL/kg/day) with risk of dehydration and electrolyte imbalance; possible presence of Clostridioides difficile or other pathogens	Marked distension with clinical significance; possible abdominal compartment syndrome progression; imaging shows moderate bowel dilation	GI bleeding with hemodynamic compromise; paralytic ileus; mesenteric ischemia signs; elevated lactate; early MODS markers
IV	Life-threatening GI failure	Complete GI failure with life-threatening complications; direct cause of multiple organ dysfunction; immediate intervention required	Massive GRV or complete intolerance to any enteral feeding; GRV measurements unreliable due to severe dysmotility; contraindication to gastric feeding	IAP > 15 mmHg with organ dysfunction; abdominal compartment syndrome by pediatric definition (sustained pressure >15 mmHg with organ dysfunction); requires decompression	Complete inability to tolerate enteral nutrition; total dependence on parenteral nutrition; EN strictly contraindicated	Intractable vomiting; high aspiration pneumonia risk; possible upper GI bleeding	Profuse diarrhea or paradoxical absence of stool (paralytic ileus); both indicate severe failure	Severe distension with abdominal compartment syndrome; tense abdomen; respiratory compromise from elevated IAP; imaging shows severe dilation or pneumatosis	Massive GI bleeding; bowel necrosis or perforation; refractory shock; sepsis from gut-origin; multiple organ failure requiring extracorporeal support

This table synthesizes pediatric-specific parameters and thresholds reported in existing literature and expert opinion for AGI severity assessment in critically ill children. The definitions integrate adjusted markers such as 5 mL/kg GRV threshold and modified energy target timelines (25% within 48 h) derived from single-center observational studies and current practice. These parameters are preliminary and require prospective multicenter validation, particularly with age-stratified norms.

AGI, acute gastrointestinal injury; FI, feeding intolerance; GRV, gastric residual volume; IAP, intra-abdominal pressure; IAH, intra-abdominal hypertension; ACS, abdominal compartment syndrome; MODS, multiple organ dysfunction syndrome; EN, enteral nutrition.

### 3.3 Prognostic value of AGI severity in pediatric critical care

Extensive evidence indicates that AGI is an independent risk factor for mortality in critically ill patients, with higher severity associated with an increased risk of death. For example, a multicenter prospective observational trial of 550 adults reported grades I–IV in 24.5, 49.4, 20.6, and 5.5%, with 60-day mortality escalating progressively (hazard ratio 1.65, 95% confidence interval 1.28–2.12) (7). Meta-analyses indicate that AGI is associated with a 33% mortality rate, with severe grades (III–IV) showing higher risk, and a relative risk (2.01, 95% confidence interval 1.20–3.37) for AGI patients compared to those without AGI (39). Stratified data reveal stark increases in higher grades, including 30.11% mortality for grade III and 61.29% for grade IV in pancreatitis patients (8). Pediatric studies, while exhibiting variable mortality rates, parallel these adult observations, revealing heightened overall mortality in severe AGI and a dose-dependent risk escalation with increasing severity (5, 7, 40). Most pediatric studies, however, did not adjust for validated severity-of-illness scores, leaving residual confounding possible; whether AGI represents an independent causal factor or a surrogate marker of overall illness severity remains unresolved. Consequently, AGI severity assessment may provide prognostic information for mortality in critically ill children, enabling risk assessment and informed clinical management, pending formal pediatric validation.

In adults, evidence from high-quality studies links higher grades to extended hospital length of stay, prolonged mechanical ventilation, and elevated MODS rates, highlighting its predictive role for poor outcomes (7). For example, grade III/IV correlated with longer mechanical ventilation (22 vs. 16 days) and increased mortality (58% vs. 28%) in critically ill COVID-19 patients (41). In studies on sepsis patients, those with AGI demonstrated significantly prolonged intensive care unit length of stay and elevated MODS incidence compared to non-AGI counterparts (42). Pediatric studies affirm comparable value: AGI trajectories in critically ill children revealed high-persistent patterns linked to longer pediatric intensive care unit length of stay and reduced enteral nutrition delivery, correlating positively with MODS. AGI occurred in 56.7% of cases, with severe grades tied to prolonged length of stay and mechanical ventilation (6). Accordingly, AGI severity provides a potentially reliable prognostic marker for hospital stay, ventilation duration, and MODS in critically ill children, supporting targeted interventions to improve outcomes, though prospective multicenter confirmation is needed.

Dynamic AGI severity assessment—tracking changes over time—significantly refines prognostic accuracy. A pivotal retrospective cohort investigation at Tianjin Children’s Hospital tracked trajectories across 642 critically ill children admitted to the pediatric intensive care unit. The research uncovered six distinct grade trajectories—low-stable, low-fluctuating, medium-decreasing, medium-increasing, high-decreasing, and high-persistent—over the critical initial 9 days post-admission. Crucially, the high-persistent group demonstrated starkly elevated 28-day (26.9%) and 60-day (32.8%) mortality rates, compounded by significantly prolonged pediatric intensive care unit length of stay and severely restricted enteral nutrition delivery. These findings underscore the vital prognostic value inherent in dynamic AGI assessment, although the single-center retrospective design and lack of external validation limit generalizability (6).

Integrating AGI severity with complementary diagnostics appears to enhance prognostic precision. In adults, combining grades with Sequential Organ Failure Assessment and Acute Physiology and Chronic Health Evaluation II scores markedly improves prediction (7, 39). Biomarkers further refine stratification; conventional markers including D-lactate, citrulline, and intestinal fatty acid-binding protein have been reported to correlate strongly with severity and clinical outcomes in adults and mixed populations (43, 44), while novel candidates show early diagnostic promise (43). However, Veldscholte et al. found that cholecystokinin (CCK), leptin, glucagon, intestinal fatty acid-binding protein 2 (I-FABP2), and citrulline had no added value in predicting enteral nutrition advancement in critically ill children (45). This negative finding highlights the need for careful evaluation of biomarker utility and suggests that biomarkers may not provide clinically meaningful predictive information for nutritional management. Discrepant results across studies likely reflect differences in patient populations, timing of measurement, and outcome definitions, emphasizing the need for standardized protocols in future biomarker research. Microbiome analysis offers a cutting-edge dimension, as metagenomic studies link AGI to gut dysbiosis, facultative anaerobe shifts, pathobiont enrichment such as Klebsiella, and altered resistome, all associated with increased mortality risk (26). Point-of-care ultrasonography enables real-time noninvasive assessment; gastric antrum metrics, intestinal wall characteristics, and superior mesenteric artery Doppler measurements have been reported to indicate severity and predict feeding intolerance (32, 46). Pediatric data, though sparser, support similar integrative approaches. Combining AGI severity with pediatric Sequential Organ Failure Assessment or Pediatric Logistic Organ Dysfunction-2 improves outcome prediction in septic children (5, 46), and biomarkers including intestinal fatty acid-binding protein and D-lactate predict AGI after traumatic brain injury (46). Point-of-care gastric ultrasound also enhances feeding tolerance assessment in critically ill children, challenging traditional gastric residual volume aspiration. Strategically combining AGI severity concepts with clinical scores, biomarkers, microbiomic data, and advanced imaging may improve overall patient prognostic accuracy, but requires prospective pediatric validation.

### 3.4 Nutritional implications of AGI severity in children

Nutritional strategies are paramount in pediatric critical care, where AGI can profoundly impair growth, immune function, and recovery, potentially leading to severe complications such as sepsis, multi-organ failure, prolonged mechanical ventilation, and elevated mortality rates (9, 47). The adult grading system, established by the ESICM Working Group, provides a standardized framework for classifying acute gastrointestinal dysfunction, with conceptual implications for nutritional management in critically ill patients (1). This framework is implicitly integrated into pediatric critical care guidelines to inform enteral nutrition strategies. For instance, the American Society for Parenteral and Enteral Nutrition (ASPEN) and Society of Critical Care Medicine (SCCM) guidelines emphasize monitoring feeding intolerance and optimizing protein delivery, aligning with AGI principles to mitigate risks of underfeeding or overfeeding (16). The European Society of Pediatric and Neonatal Intensive Care (ESPNIC) position statement further reflects AGI influence by recommending tailored nutrition support based on gastrointestinal symptom severity, consistent with severity-based interventions (15). Recent expert consensus guidelines, such as those from the French Intensive Care Society (SRLF) and the French-Speaking Group of Pediatric Emergency Physicians and Intensivists (GFRUP), reinforce the importance of individualized nutritional approaches, echoing the grading system’s role in stratifying patients by severity to improve outcomes in critically ill children (48). Collectively, severity assessment concepts underpin evidence-based nutritional decision-making, promoting safer and more effective nutrient delivery in this vulnerable population.

In critically ill children, severity assessment spanning from grade I (risk of dysfunction) to grade IV (gastrointestinal failure with distant organ involvement) offers a conceptual framework for guiding the initiation, titration, or suspension of enteral nutrition, ensuring customized support that matches gastrointestinal tolerance and physiological demands. In grades 0–II, indicative of mild or no feeding intolerance, early and aggressive enteral nutrition is advocated to achieve caloric targets, as evidence from pediatric cohorts demonstrates improved energy delivery and reduced length of stay without exacerbating gastrointestinal stress (6). For instance, observational studies in neonatal and pediatric intensive care unit settings report that timely enteral nutrition in low-grade AGI correlates with lower infection rates and better nitrogen balance (5, 49). Conversely, in grades III–IV, nutritional approaches shift toward caution to avert translocation and sepsis. Here, trophic enteral nutrition combined with parenteral nutrition is preferred, with progression monitoring dictating escalation. A multicenter analysis revealed that persistent high-grade trajectories in children halve enteral nutrition tolerance, underscoring the need for dynamic reassessment every 24–48 h to optimize parenteral nutrition-to-enteral nutrition ratios (50). A severity-based nutritional synthesis is presented in Table 1.

**Figure 2:**
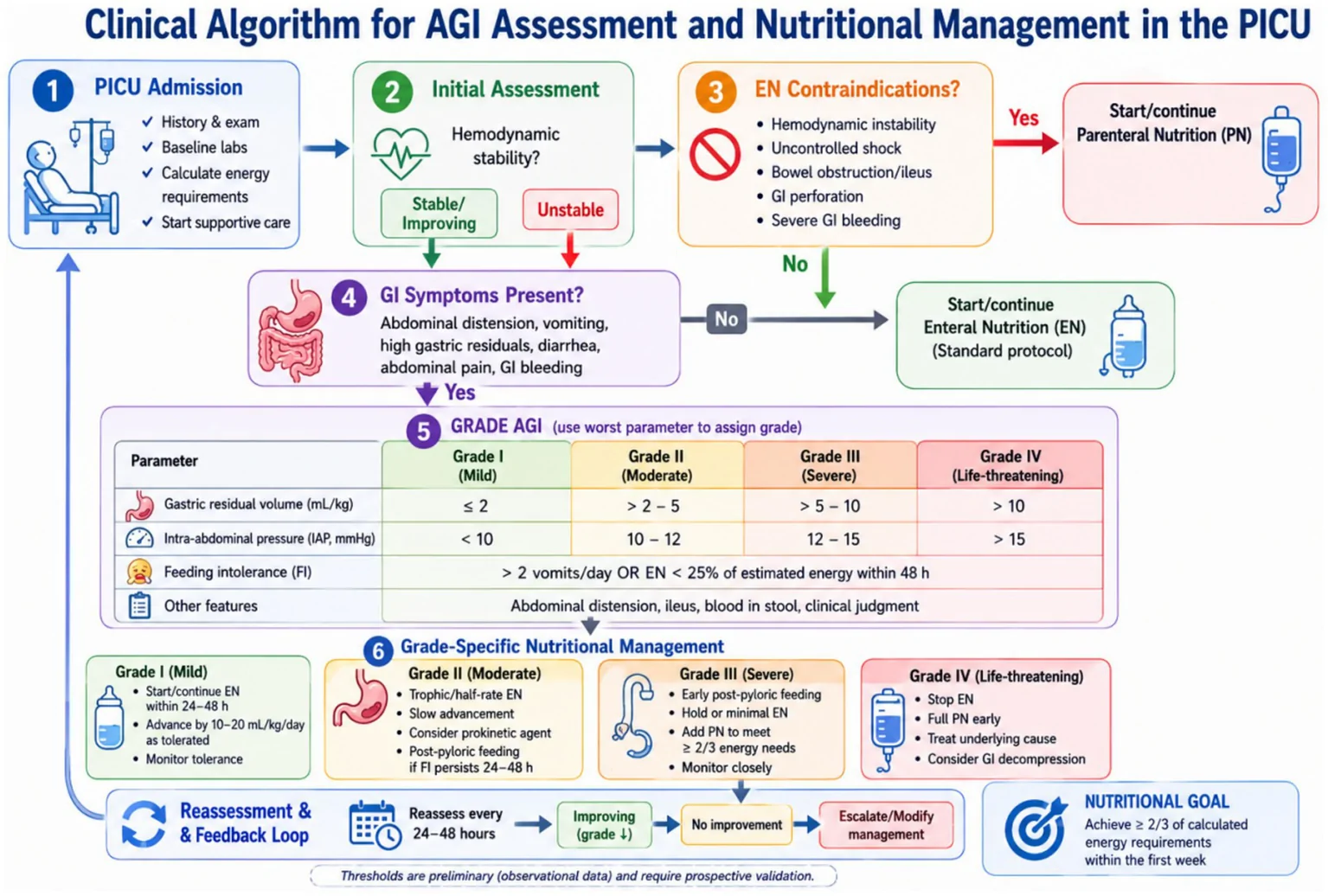
Conceptual clinical workflow for AGI severity assessment and nutritional management in the pediatric intensive care unit (PICU), synthesized from existing evidence and guideline recommendations. Upon admission, patients are stratified by hemodynamic stability and enteral nutrition (EN) contraindications. In the presence of gastrointestinal symptoms, patients are assessed using pediatric-adapted severity criteria derived from existing literature. Severity-based concepts guide EN titration, prokinetic or post-pyloric feeding for feeding intolerance (FI), and parenteral nutrition (PN) integration for severe failure (grades III–IV). A continuous feedback loop mandates reassessment every 24–48 h. The nutritional goal is to achieve at least two-thirds of calculated energy requirements within the first week.

Evidence on enteral nutrition benefits in high-grade AGI (III–IV) remains equivocal. While some observational studies suggest improved outcomes with trophic enteral nutrition, the risk of aspiration, bacterial translocation, and intra-abdominal hypertension complications must be carefully weighed. The absence of pediatric randomized controlled trials in this population precludes definitive recommendations. Current practice is guided by expert consensus, which recommends cautious trophic enteral nutrition with parenteral supplementation, but this approach requires prospective validation. To operationalize these principles, a structured conceptual workflow (Figure 2) synthesizes assessment with nutritional decision trees based on existing evidence and guideline recommendations, illustrating how multidisciplinary teams might navigate the temporal flux of severity trajectories. This visual heuristic is intended to streamline bedside workflows and safeguard nutritional autonomy in critically ill children, while acknowledging that it represents a conceptual synthesis rather than a validated protocol.

## 4 Discussion

### 4.1 Critical appraisal of current evidence

Despite its potential for prognostic evaluation and nutritional guidance, significant implementation challenges limit clinical adoption of AGI severity assessment. Although the prognostic value of AGI severity is increasingly recognized, clinical adoption remains limited by the absence of validated pediatric-specific tools. Central to this gap is subjectivity, intensified in pediatrics by children’s inability to articulate symptoms and their heterogeneous physiology. Severity classification hinges on clinician acumen, creating interobserver variance.

Critically, the association between higher AGI grades and mortality warrants careful interpretation regarding causality. Current evidence does not definitively establish whether AGI represents an independent causal factor influencing outcomes or merely a surrogate marker of overall disease severity. Several confounding factors may explain the observed associations: sicker patients with more severe underlying conditions are more likely to develop gastrointestinal dysfunction; higher AGI grades may correlate with greater organ failure burden rather than exerting independent pathogenic effects; and the use of vasoactive medications and sedatives in more severely ill patients may independently impair gastrointestinal function. These confounders have not been systematically addressed in pediatric studies, limiting causal inference.

Feeding intolerance, a key marker, fuses subjective harbingers—vomiting, distension, residuals, and bleeding—eliciting inter-rater divergence. Bowel sounds fare no better: even among experienced intensivists, reliability is modest due to absent auscultation standards. In ventilated or sedated children, confounders including agitation or neuromuscular blockade distort evaluations, rendering objective grading elusive. These limitations hinder prognostic accuracy and impede nutritional guidance, as delayed assignment may prolong fasting or escalate parenteral nutrition risks.

The recent GASTRIC-PICU Trial (17) adds further complexity to this discussion. This large multicenter trial demonstrated non-inferiority of not routinely measuring GRV compared to every-6-h monitoring, with significantly improved energy delivery in the no-routine-measurement group. This finding directly challenges the fundamental assumption underlying GRV-based AGI assessment—that routine GRV measurement is necessary for safe and effective enteral nutrition. Future pediatric AGI severity criteria may need to reduce or eliminate reliance on GRV, focusing instead on clinical signs of feeding intolerance, ultrasound assessment, or other objective markers.

Adapting severity assessment to children requires more than adult extrapolation, given profound developmental differences in anatomy, physiology, immunology, disease spectra, and compensatory capacities. The principal contention is the paucity of uniform standards for assessing gastrointestinal symptoms in pediatric populations. Diarrhea defies uniformity; infants’ loose stools reflect immature flora versus pathological dysmotility in older children, confounding severity assessment without age-stratified norms. Vomiting is amplified in young infants due to horizontal gastric positioning and weak lower esophageal sphincter tone, potentially misattributing benign reflux to severity escalation. Gastric residual volume lacks consensus on frequency and thresholds, and the GASTRIC-PICU trial has now shown that routine scheduled measurement confers no benefit (17). Nutritional guidance amplifies these challenges. Evidence on enteral nutrition benefits in high-grade AGI (III–IV) remains equivocal, with mixed outcomes on mortality and infections versus aspiration risks (51). Prokinetic efficacy remains uncertain, with sparse randomized evidence; the post-pyloric versus gastric feeding debate remains unresolved. Age-specific caloric and protein targets remain undefined, complicating personalized plans.

Heterogeneity across pediatric populations further complicates evidence interpretation. Studies include widely varying age ranges from neonates to adolescents without stratification, despite profound physiological differences that likely affect AGI development, progression, and response to nutritional interventions. Single-center studies from different geographic regions exhibit varying patient demographics, disease spectra, and practice patterns, limiting generalizability. Furthermore, the inconsistency between observational studies regarding the prognostic value of specific AGI components (particularly GRV monitoring) underscores the current uncertainty in the evidence base.

### 4.2 Limitations and methodological gaps

The pediatric evidence base remains constrained by a scarcity of high-quality investigations. Predominantly small-scale and observational, these studies—encompassing fewer than 10 pediatric randomized controlled trials—impede causal inference and engender confounding biases. Single-center retrospective analyses exhibit heterogeneity, amplifying regional disparities without robust multinational multicenter trials.

Publication bias likely inflates the apparent strength of AGI-prognosis associations, as studies reporting null findings may remain unpublished. Furthermore, external validity is compromised by the narrow patient populations typically studied in single centers with specific practice patterns. The variability in pediatric age groups, as mentioned above, is rarely addressed through stratification.

The absence of standardized AGI definitions across studies limits comparability. While the ESICM framework provides adult standards, pediatric studies have applied varied thresholds for GRV, IAP, and feeding intolerance, introducing heterogeneity that complicates evidence synthesis. A substantial proportion of published AGI studies have been conducted in China, raising questions about generalizability to diverse populations with different disease spectra, nutritional practices, and healthcare systems. Multi-center validation studies incorporating geographically diverse populations are urgently needed to establish external validity.

The absence of age-stratified norms for AGI assessment parameters represents a critical gap. Thresholds developed for older children may not apply to neonates with immature gastrointestinal motility or adolescents with near-adult physiology. For example, applying a uniform GRV threshold of 5 mL/kg from neonates to adolescents ignores developmental differences in gastric capacity, emptying rates, and feeding volumes. Similarly, IAP thresholds derived from mixed-age cohorts may not account for age-related differences in abdominal wall compliance and intra-abdominal volume. Future research must establish age-specific reference ranges across developmental stages to enable accurate severity assessment.

Thus, rather than proposing a new clinical framework, this review identifies prerequisites for future development: formal consensus methodology involving multidisciplinary pediatric stakeholders, systematic evidence synthesis through prospective multicenter validation, biomarker integration, point-of-care ultrasound standardization, and age-stratified nutritional trials.

### 4.3 Future directions and research priorities

AGI severity assessment holds conceptual promise for identifying high-risk patients and guiding nutrition in pediatric intensive care units, yet its clinical adoption requires multicenter randomized controlled trials and validated pediatric-specific tools to enhance applicability. This review underscores that current pediatric adaptations of adult AGI criteria remain preliminary and should not be implemented as standardized clinical frameworks until rigorous validation is achieved. Future research should prioritize formal consensus development using modified Delphi or nominal group techniques with international pediatric critical care societies, followed by prospective multicenter validation of age-specific thresholds for gastric residual volume, intra-abdominal pressure, and feeding intolerance. Integration of biomarkers (D-lactate, citrulline, intestinal fatty acid-binding protein) and point-of-care gastric ultrasound into severity algorithms requires standardized protocols and outcome-linked validation. Age-stratified nutritional trials comparing aggressive early enteral nutrition versus trophic feeding plus parenteral nutrition across AGI severity grades are essential to define evidence-based, personalized nutritional strategies in pediatric critical care.

Several conceptual issues merit further scientific interpretation. First, the relationship between AGI and poor outcomes remains mechanistically unclear: is AGI an independent driver of organ dysfunction via bacterial translocation, systemic inflammation, and immune dysregulation, or merely a marker of underlying disease severity? Answering this question has profound implications for whether AGI-directed interventions could improve outcomes independently of treating the underlying critical illness. The ‘gut hypothesis’ of MODS suggests a causal role, but definitive evidence from pediatric interventional trials is lacking.

Second, the dynamic nature of AGI severity—with trajectories that may improve, worsen, or remain stable—offers opportunities for precision nutrition. Patients with high-persistent trajectories might benefit from more aggressive parenteral support with trophic enteral nutrition, whereas those with improving trajectories could be candidates for accelerated enteral advancement. However, current evidence derives primarily from a single-center retrospective study (6), and prospective validation is essential before clinical implementation.

Third, emerging technologies may address current limitations. Biomarkers such as I-FABP2, citrulline, and D-lactate show promise for objective severity assessment; however, Veldscholte et al. (JPGN 2023) found that CCK, leptin, glucagon, I-FABP2, and citrulline had no added value in predicting enteral nutrition advancement in critically ill children, highlighting the need for careful evaluation of biomarker utility (45). Microbiomic analysis offers a cutting-edge dimension for understanding dysbiosis and its role in AGI pathophysiology (52, 53). Point-of-care gastric ultrasound represents a non-invasive alternative to GRV aspiration that may enhance feeding tolerance assessment. However, standardization and validation of these technologies in pediatric populations are required before integration into severity algorithms.

Fourth, international consensus development using modified Delphi or nominal group techniques with pediatric critical care societies could establish standardized definitions and thresholds, reducing current subjectivity. These consensus-based frameworks could then undergo prospective validation in multicenter cohorts.

## 5 Conclusion

The acute gastrointestinal injury grading concept identifies high-risk patients and informs nutritional decision-making, but requires multicenter validation and pediatric-specific tools before clinical adoption. Higher AGI severity consistently associates with increased mortality, prolonged mechanical ventilation, and extended lengths of stay. Dynamic trajectories—particularly high-persistent patterns—refine prognostic precision beyond single assessments. This review synthesizes existing pediatric adaptations of adult criteria, including observationally derived gastric residual volume thresholds (>5 mL/kg), intra-abdominal pressure gradations (10–12 mmHg for grade II; 12–15 mmHg for grade III; >15 mmHg for grade IV), and feeding intolerance criteria (>2 vomits/day or enteral nutrition <25% of calories within 48 h); all of these parameters remain candidates for validation rather than established standards. Nutritionally, grades 0–II support early aggressive enteral nutrition, whereas grades III–IV warrant trophic enteral nutrition combined with parenteral nutrition, reassessed every 24–48 h. Subjective variables, incomplete validation, and scarce pediatric randomized controlled trials remain barriers. Future research should prioritize international consensus development using formal Delphi or nominal-group methodology, multicenter validation, biomarker integration, point-of-care ultrasound, and age-stratified nutritional trials to standardize this framework.
