# Correction: Acute gastrointestinal injury severity assessment informs nutritional support and prognostic evaluation in critically ill children: a narrative review

**DOI:** 10.3389/fnut.2026.1989398

**Published:** 2026-09-23

**Authors:** Cizheng Zeng, Tufeng Li, Jiayuan Wu, Shiwen Rong, Xiaohua Liu, Yinhui Chen, Nali Cai, Limei Zhang, Wen Li, Ling Liu

**Affiliations:** 1 Pediatric Intensive Care Unit, Affiliated Hospital of Guangdong Medical University, Zhanjiang, China; 2 Department of Scientific Research, Affiliated Hospital of Guangdong Medical University, Zhanjiang, China

**Keywords:** critical illness, enteral feeding, feeding intolerance, gastric residual volume, intra-abdominal hypertension, multiple organ dysfunction, nutritional support, pediatric intensive care

In the published article, there was an error in the **Funding** statement. The funding name and number were omitted from the final published manuscript. The correct **Funding** statement appears below.

