# Supplementary material for: Acute gastrointestinal injury severity assessment informs nutritional support and prognostic evaluation in critically ill children: a narrative review
Source: Front Nutr. 2026 Aug 14;13:1911973. doi: 10.3389/fnut.2026.1911973 (PMC13521867; doi:10.3389/fnut.2026.1911973)
Supplement: Supplementary file 1 [file Supplementary_file_1.docx]

**Supplementary Materials**

**Figure S1. PRISMA-style flow diagram of literature selection process for the narrative review.**

**Identification:** Records identified through database searching (n = 1,247): PubMed (n = 312), Embase (n = 278), Web of Science (n = 245), Google Scholar (n = 186), Cochrane Library (n = 72), CINAHL (n = 65), CNKI (n = 54), Wanfang Data (n = 35). Additional records identified through citation searching (n = 78). **Screening:** Records after duplicates removed (n = 1,242). Records screened (n = 1,242). Records excluded based on title/abstract screening (n = 1,018). **Eligibility:** Full-text articles assessed for eligibility (n = 224). Full-text articles excluded (n = 146): Insufficient pediatric-specific data (n = 58); Absence of AGI severity assessment (n = 42); Low-quality methodology (n = 28); Conference abstracts (n = 12); Non-English without translation (n = 6). **Included:** Studies included in narrative review (n = 78): Original research articles (n = 52); Guidelines/consensus statements (n = 16); Reviews/meta-analyses (n = 10).
